# Esophageal Cancer Staging in Malawi: The Feasibility of Chest Radiography and Abdominal Ultrasound for Initial Evaluation

**DOI:** 10.21203/rs.3.rs-6994944/v1

**Published:** 2025-08-05

**Authors:** Brittney M. Williams, Gift Mulima, Bongani Kaimila, Katherine Drew Marapese, Ande W. Salima, Austin Evans, Natasha Ngwira, Chifundo Kajombo, Jared Gallaher, Anthony Charles, Katrina McGinty, Geoffrey Buckle, Gita N. Mody

**Affiliations:** Emory University; Kamuzu Central Hospital; Kamuzu Central Hospital; University of North Carolina; Kamuzu Central Hospital; University of North Carolina; Kamuzu Central Hospital; Kamuzu Central Hospital; University of North Carolina; University of Vermont; Novant Health; University of California San Francisco (UCSF); University of North Carolina

**Keywords:** Esophageal cancer, staging, low- and middle-income countries

## Abstract

**Background:**

Esophageal cancer (EC) is the third leading cause of cancer-related morbidity and mortality in Malawi. Given limited imaging capacity and high costs, staging is not routinely performed. One proposed staging algorithm is to first evaluate for metastatic disease using low-cost chest radiography (CXR) and abdominal ultrasound (US) followed by confirmatory computerized tomography (CT) of the chest and abdomen if no metastases identified on initial screening. The feasibility of this approach is unknown for EC in sub–Saharan Africa and was studied in the context of a larger prospective observational cohort study of EC in Malawi.

**Methods:**

From 2021 to 2022, EC patients at Kamuzu Central Hospital in Lilongwe, Malawi enrolled in the Treatment Outcomes of Esophageal Cancer in Malawi (TOEC-M) study were recruited. Participants were scheduled for a CXR, US, and CT scan as part of this sub-study. Participant characteristics, completion rates, imaging findings, and barriers to completion were documented. For participants undergoing all three imaging studies, sensitivity and specificity were calculated.

**Results:**

Of 150 patients in TOEC-M, 67 (44.7%) enrolled in this sub-study. Mean age was 55.4 years and 50.8% were males. The majority had mid-esophageal (38 [56.7%]) squamous cell carcinomas (54 [80.6%]). CXR was completed in 54 (80.6%) study participants, US in 43 (64.2%), CT chest in 29 (43.3%), and CT abdomen in 24 (35.8%). Sixteen (23.9%) completed all studies and 4 (6.0%) did not undergo any imaging. Of the 63 patients that were imaged, metastatic disease was identified in 18 (28.6%) by any modality. Positive findings were identified on 3 (5.6%) CXRs, 4 (9.3%) US, and 18 (62.1%) CTs, most frequently liver masses followed by lung nodules and adenopathy. Barriers to imaging completion included participant functional status and scanner availability.

**Conclusions:**

As access to EC treatment modalities expands, feasible and accurate staging will become increasingly important to guide clinical management. Our results suggest that CXR and US may serve as useful initial tools for assessing metastatic disease. In patients not medically fit for oncologic treatment, positive findings on CXR and US may allow CT to be deferred. Barriers to implementation of a pragmatic stepwise staging algorithm identified in this study can inform future research and care for patients with EC in similar resource-limited settings.

## Background

Esophageal cancer (EC) is a leading cause of global cancer-related morbidity and mortality. EC is currently the eighth most commonly diagnosed and the sixth most common cause of cancer death worldwide [1]. The incidence of EC varies widely across geographic regions with an estimated 80% of cases and deaths occurring in low- and middle-income countries (LMIC) [2]. Eastern Africa, where esophageal squamous cell carcinoma (ESCC) is the predominant histology, has been identified as one of several distinct regions with a high burden of EC incidence and mortality [3, 4]. Malawi, a country of 21.1 million population, has the third highest EC mortality rate worldwide at 19.5 male patients per 100,000 [1].

Despite this geographic preponderance, there remains a critical knowledge gap regarding the optimal method of diagnosis, staging, and treatment of EC in this region. In this setting, more than 90% of patients present with advanced disease and there is limited access to treatment with curative intent [5]. In Malawi, treatment options are currently palliative, consisting of endoscopic stent placement and chemotherapy. However, as treatment options expand in Malawi and other resource-limited settings, accurate staging will become increasingly important for appropriate triage, optimal resource use, and to guide evidence-based care. Additionally, the presenting stage of esophageal cancer in Malawi has not previously been described.

In recognition of these gaps, there has been a global call to action for the study of EC in eastern and southern Africa by the African Esophageal Cancer Consortium (AfrECC) [6]. AfrECC was founded to advance research, training, and collaborative efforts focused on esophageal cancer control in eastern and southern Africa [6, 7]. UNC Project-Malawi was a founding institutional member of the consortium. Aligned with this agenda, this study aims to evaluate disease stage among patients with esophageal cancer presenting to a national cancer referral hospital in Malawi, and the diagnostic performance of low-cost diagnostic imaging for staging during initial work up. The study also aims to evaluate barriers to completing staging workup and the feasibility of obtaining chest radiograph (CXR), abdominal ultrasound (US), and computed tomography (CT) scans. These insights will help address key knowledge and implementation gaps to support evidence-based clinical practice and inform healthy policy.

ESCC is staged using the tumor-node-metastasis (TNM) classification system [8]. Leading guidelines from high-income countries, including those issued by the National Comprehensive Cancer Network (NCCN) and the Society of Thoracic Surgeons [9], recommend initial staging of esophageal cancer using contrast-enhanced CT of the chest and abdomen; pelvic CT as clinically indicated; FDG-PET/CT if no known metastatic disease; and endoscopic ultrasound (EUS) [9]. The NCCN Harmonized Guidelines^™^ for Sub-Saharan Africa for Esophageal and Esophagogastric Junction Cancers, endorsed by the Government of Malawi, recommend a resource-adapted approach for staging that includes CT of the chest and abdomen, with pelvic CT as clinically indicated. This recommendation is categorized within the guidelines as a “generally available standard of care.” However, in Malawi and many other resource-limited settings, access to CT scans is often limited by cost, availability, and infrastructure constraints— and access to advanced modalities such as PET/CT and EUS is virtually nonexistent.

**Figure 1:**
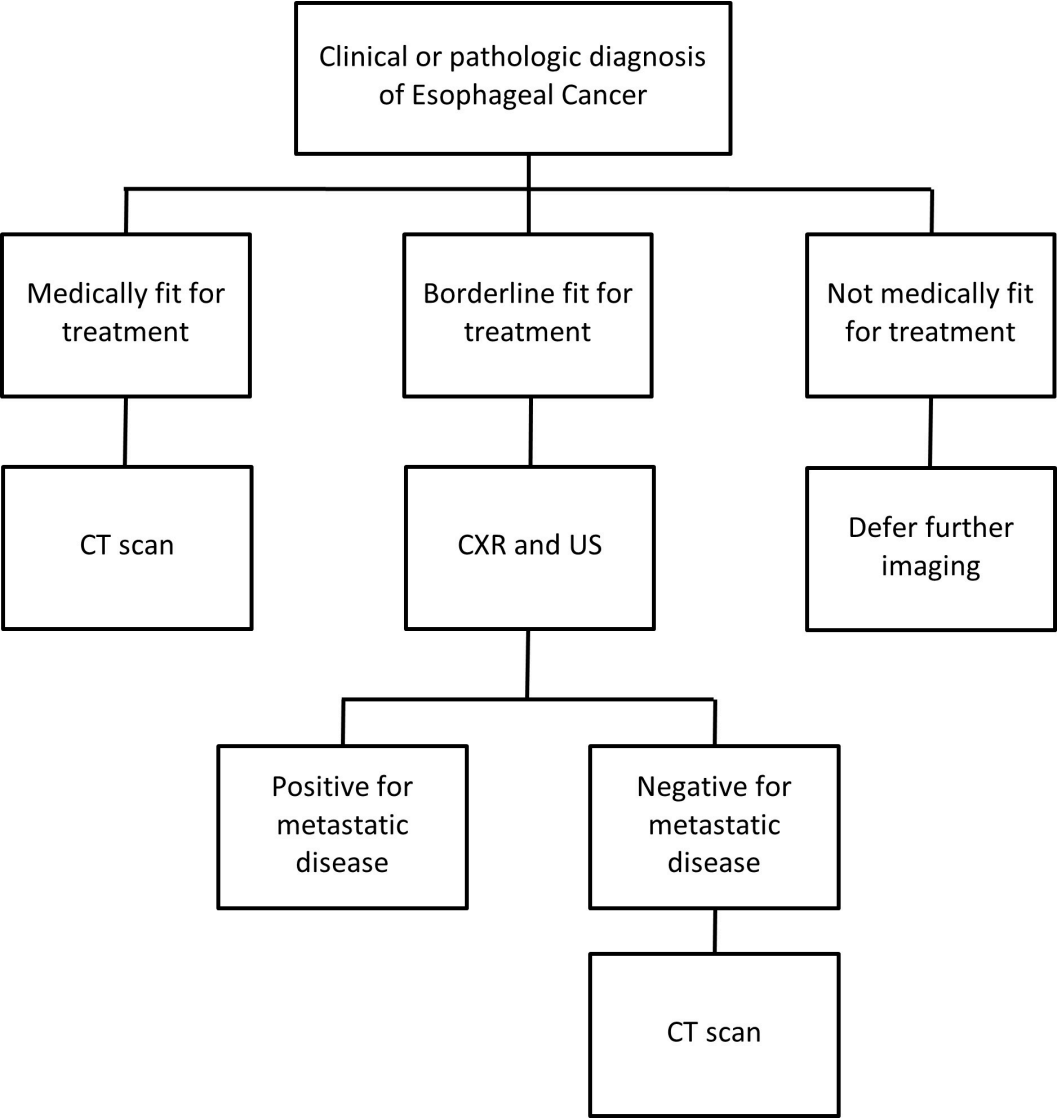
Proposed algorithm for staging esophageal cancer patients in Malawi.

Given the challenges associated with cancer staging in LMICs, an alternative classification system has been proposed for = resource-limited settings by the Union for International Cancer Control. The Essential TNM system utilizes the same classification components of the standard TNM system but instead begins with assessment of distant metastasis before further diagnostic assessments [10]. This sequence represents a more pragmatic approach in resource-limiting settings, where identifying metastatic disease upfront can obviate the need for further testing. While not previously tailored to esophageal cancer, these principles informed the development of our proposed staging algorithm (Fig. 1). We hypothesize that CXR and abdominal US with confirmatory CT of the chest and abdomen can be utilized as a low-cost, pragmatic approach for initial assessment for metastatic disease in ESCC in Malawi. A staging algorithm has the potential to guide clinical decision making and optimize resource utilization. We hypothesize that a sequential staging approach using CXR and US, with CT of the chest/abdomen only if initial evaluates are negative can be utilized as a low cost, pragmatic approach

## Methods

### Study Design and Setting

This study was performed as part of a larger prospective observational cohort study Treatment Outcomes of Esophageal Cancer in Malawi (TOEC-M) (NCT05177393). The parent study was retrospectively registered with the ClinicalTrials.gov database on December 15, 2021. Patients with either a pathologically or endoscopically confirmed diagnosis of EC were prospectively enrolled from Kamuzu Central Hospital (KCH), an 800-bed tertiary care hospital in Lilongwe, Malawi that serves 8 districts in the central region of Malawi with a catchment area of approximately 6 million people.

### Study Participants and Data Collection

Methods of recruitment, ascertainment, and informed consent are previously described elsewhere [7]. Inclusion criteria were patients over 18 years of age with either a pathologically confirmed or presumptive diagnosis of EC based on barium swallow or endoscopy and clinical stability to independently present to a radiology facility. Patients that were pregnant, had another known cancer diagnosis, or were already receiving EC treatment were excluded. Informed consent was taken for both the parent study and the sub-study. Costs of imaging studies were covered by the study.

Baseline socio-demographic and clinical information was collected upon enrollment, including length of symptoms, endoscopic findings, and histology if available. Each participant was scheduled for CXR, abdominal US, and CT of the chest and abdomen to evaluate for evidence of metastatic disease. During the course of the study, the CT facilities at KCH were intermittently not available, leading to an effort to provide transportation to private facilities using available funds.

Imaging studies were interpreted by trained radiologists at KCH with support from the University of North Carolina. Positive findings were defined as the presence of pulmonary nodules, pleural effusions, hepatic masses, or ascites. The aim of our study was to determine to examine sensitivity and specificity of CXR/US to set the stage for testing and evaluation of a pragmatic algorithm. The long term goal of this study was to inform our understanding of the feasibility and limitations of sequential, algorithm staging approaches for EC.

### Statistical Analysis

Descriptive statistics were used to describe the baseline characteristics of our patient population, compliance rates with each imaging study, and imaging study results. Differences in characteristics between eligible participants that did and did not enroll into the sub-study were compared using Pearson’s Chi-squared test. Sensitivity and specificity of CXR and US for detecting distant metastasis were calculated by generating a 2x2 table. CT scan was considered the ‘gold standard’ as PET/CT is unavailable in this setting. Barriers to completion of imaging studies were identified during weekly meetings with the research team.

## Results

Of 150 total eligible patients in TOEC-M, 67 (44.7%) enrolled in the staging sub-study. Mean age was 55.4 years and 50.8% were males. Participants were largely never smokers (70.2%) and had no history of alcohol abuse (70.2%). Only 6 (9.0%) patients were insured. There were no significant differences between characteristics of patients enrolled and not enrolled into the sub-study (Table 1).

The majority of participants had mid-esophageal tumors (38 [56.7%]). Endoscopic biopsies were obtained in 60 (89.6%) patients, of which squamous cell carcinoma was the most prevalent histology (54 [90%]). The median duration of symptoms was 5 months (IQR 3–6) (Table 2). CXR was completed in n = 54 (80.6%), right upper quadrant US in n = 43 (64.2%), CT chest in n = 29 (43.3%), and CT abdomen in n = 24 (35.8%). All studies were completed in 16 (23.9%) and only 4 (6.0%) did not undergo any imaging (Table 3).

Of the 63 patients that were imaged, metastatic disease was identified in 18 (28.6%) by any modality. Positive findings consistent with metastasis were identified on 3 (5.6%) CXRs, 4 (9.3%) US, and 18 (62.1%) CTs. The most frequent sites of metastasis were liver followed by lung and distant lymph nodes (Table 4). None of the participants with positive findings on CXR and/or US underwent confirmatory CT precluding our ability to calculate false positive rate.

Barriers to imaging completion included symptom burden and facility availability including malfunctioning scanners and distance to private facilities. Notably, in the group of 16 patients that underwent all 4 imaging modalities (Table 5), CXR and/or US did not detect any metastases, including among the 7 patients who were found to have metastasis on CT, leading to sensitivity of 0% in this small sample. CT chest/abdomen also did not show metastases in n = 9, leading to a specificity of 100% of CXR/US and a negative predictive value of 56.3%.

## Discussion

We conducted a prospective observational cohort study examining the feasibility of using low-cost imaging modalities to stage the EC population in Malawi, a setting where staging of EC is not routinely performed. Of our potential participant pool of 150 patients, 67 (44.7%) patients were eligible and enrolled to receive imaging for staging. We found that the majority of patients had mid-esophageal squamous cell carcinomas and that 18 (28.6%) had evidence of metastatic disease. CXR and abdominal US were feasible to obtain and interpret as a low-cost solution for initial evaluation for metastases in resource-limited environments. As the sample size was limited, and we were unable to calculate the sensitivity of these imaging studies alone, they did show excellent specificity and may be used to defer CT scan if positive.

Previous studies in high-income countries have shown the sensitivity of abdominal US and CXR in detecting metastatic disease in EC patients to be 65% and 68%, respectively [11]. Given a presumably higher prevalence of advanced disease in our East African population, the sensitivity of these studies is expected to be higher. While not yet validated, CXR and US have been previously used in resource-limited settings to evaluate the presence of metastatic disease. For example, Tenwek hospital in Kenya used CXR and abdominal US to exclude metastatic disease prior to esophagectomy [12]. As access to curative treatment options expands across this region, an algorithm for pre-treatment evaluation may be useful to guide resource allocation and clinical management, particularly when cost of cross-sectional imaging studies remains prohibitive to most patients.

Our study did have several limitations largely related to our small sample size and limited completion rates. We faced several obstacles to imaging completion due to availability of radiology facilities, transportation, and the frailty of our population. Coordination with radiology was also a key barrier to completion given the limited availability of equipment. While it was our hope that all three studies could be performed on the same day to enhance completion rates, there was often discontinuity in this workflow leading to long patient wait times and difficulty rescheduling. Symptom burden (as patients were very ill due to obstructing esophageal tumors) often impeded patients from withstanding multi-day tests.

## Conclusions

In Malawi and similar resource-limited settings, CXR and abdominal US are feasible, low-cost modalities as an initial evaluation for metastatic disease in EC patients. However, given barriers to completion of multiple imaging studies, focusing on obtaining or lowering costs of CT scans should be prioritized, particularly in patients medically fit for further treatment, e.g. chemotherapy or radiotherapy. For patients with borderline fitness for treatment, CXR and US can be used for initial staging as positive studies could potentially defer need for CT scan. In those too unwell for treatment, imaging could be deferred altogether. Future work on cost-effectiveness and applicability outside of a hospital-based population will be required prior to implementation.

## Figures and Tables

**Table 1:** Demographics of TOEC-M eligible patients by enrollment in the imaging sub-study

	Enrolled (N = 67)	Not Enrolled (N = 83)	Total (N = 150)	p
Age (years), mean (SD)	55.4 (12.6)	54.8 (3.6)	55.1 (13.1)	0.79
Male, n (%)	34 (50.8)	46 (55.4)	80 (53.3)	0.57
HIV positive, n (%)	7 (10.5)	12 (14.5)	19 (12.7)	0.13
Smoking history, n (%)				0.92
Current	4 (6.0)	6 (7.2)	10 (6.7)	
Former	16 (23.9)	18 (21.7)	34 (22.7)	
Never	47 (70.2)	59. (71.1)	106 (70.7)	
Alcohol history, n (%)				0.78
Current	3 (4.5)	6 (7.3)	9 (6.0)	
Former	17 (25.4)	19 (23.2)	36 (24.2)	
Never	47 (70.2)	57 (69.5)	104 (69.8)	
Highest level of education, n (%)				0.69
None	35 (52.2)	44 (53.0)	79 (52.7)	
Primary	21 (31.3)	20 (24.1)	41 (27.3)	
Secondary	9 (13.4)	16 (19.3)	25 (16.7)	
Technical college or University	2 (3.0)	3 (3.6)	5 (3.3)	
Income (kwacha/USD) per year, median (IQR)	1350000/777 (500000/288-2000000/1152)	1250000/720 (600000/345-2500000/1439)	1350000/777 (500000/288-2000000/1132)	0.75
Insured, n (%)	6 (9.0)	4 (4.9)	10 (6.8)	0.35

**Table 2:** Tumor characteristics of TOEC-M eligible patients by enrollment in the imaging sub-study

	Enrolled (N = 67)	Not Enrolled (N = 83)	Total (N = 150)	p
Tumor location, n (%)				0.17
Upper	8 (11.9)	19 (22.9)	27 (18.0)	
Middle	38 (56.7)	34 (41.0)	72 (48.0)	
Distal	8 (11.9)	9 (10.8)	17 (11.3)	
Missing	13 (19.4)	21 (25.3)	34 (22.7)	
Tumor length (cm), median (IQR)	7 (5–8)	6 (5–7)	6 (5–8)	0.31
Mode of diagnosis, n (%)				0.17
Biopsy	60 (89.6)	66 (79.5)	126 (84.0)	
Endoscopy without biopsy	7 (10.5)	14 (16.9)	21 (14.0)	
Endoscopy with non-confirmatory biopsy	0 (0)	3 (3.6)	3 (2.0)	
Biopsy results, n (%)				0.28
Squamous cell carcinoma	54 (80.6)	56 (67.5)	110 (73.3)	
Adenocarcinoma	0 (0)	1 (1.2)	1 (0.7)	
Other malignancy	1 (1.5)	3 (3.6)	4 (2.7)	
Dysplasia	1 (1.5)	3 (3.6)	4 (2.7)	
Benign	2 (3.0)	4 (4.8)	6 (4.0)	
Non-diagnostic	2 (3.0)	0 (0)	2 (1.3)	
Duration of symptoms (months), median (IQR)	5 (3–6)	4 (3–6)	4 (3–6)	0.25

**Table 3:** Imaging completion rates (N = 67)

	Completed, n (%)
None	4 (6.0)
Chest x-ray	54 (80.6)
Abdominal ultrasound	43 (64.2)
CT chest	29 (43.3)
CT abdomen	24 (35.8)
Chest X-ray, Abdominal US, and CT chest and abdomen	16 (23.9)

**Table 4:** Imaging Findings*

	CXR (n = 54)	US (n = 43)	CT (n = 29)
Metastatic disease, n (%)	3 (5.6)	4 (9.3)	18 (62.1)
Liver	-	4 (9.3)	5 (17.2)
Lungs	2 (3.7)	-	3 (10.3)
Adenopathy	0 (0)	0 (0)	3 (10.3)
Bones	0 (0)	-	2 (6.9)
Other	0 (0)	0 (0)	5 (17.2)
Missing	1 (1.9)	0 (0)	0 (0)

* More than one imaging test may have been completed in each participant

**Table 5:** Rates of imaging detection of metastatic disease (n = 16) in patients undergoing all 4 imaging modalities

	CT positive	CT negative
CXR or US positive	0	0
CXR and US negative	7	9

## Data Availability

The datasets used and/or analysed during the current study are available from the corresponding author on reasonable request.

## Abbreviations

EC: Esophageal cancer
ESCC: Esophageal squamous cell carcinoma
CT: Computed tomography
CXR: Chest radiography
KCH: Kamuzu Central Hospital
LMIC: Low- and middle-income countries
PET: Positron emission tomography
TNM: Tumor-node-metastasis
TOEC-M: Treatment Outcomes of Esophageal Cancer in Malawi
US: Ultrasound
